# Bibliometric and social network analysis of a Clinical and Translational Resource awardee: An Oklahoma experience 2014–2021

**DOI:** 10.1017/cts.2023.690

**Published:** 2023-12-07

**Authors:** Janis E. Campbell, Motolani E. Ogunsanya, Nicole Holmes, Tim VanWagoner, Judith James

**Affiliations:** 1 Department of Biostatistics and Epidemiology, Hudson College of Public Health, University of Oklahoma Health Sciences Center, Oklahoma City, OK, USA; 2 College of Pharmacy, The University of Oklahoma Health Sciences Center, Oklahoma City, OK, USA; 3 TSET Health Promotion Research Center, Stephenson Cancer Center, University of Oklahoma Health Sciences Center, Oklahoma City, OK, USA; 4 Oklahoma Clinical and Translational Science, University of Oklahoma Health Sciences Center, Oklahoma City, OK, USA; 5 University of Oklahoma Health Sciences Center, Oklahoma City, OK, USA

**Keywords:** Social network analysis, co-authorship, clinical and translational research, evaluation, bibliometric

## Abstract

**Background:**

Social Network Analysis is a method of analyzing coauthorship networks or relationships through graph theory. Institutional Development Award (IDeA) Networks for Clinical and Translational Research (IDeA-CTR) was designed to expand the capability for clinical and translational research to enhance National Institutes of Health funding.

**Methods:**

All publications from a cohort of clinical and translational scientists in Oklahoma were collected through a PubMed search for 2014 through 2021 in October 2022. For this study’s bibliometric portion, we pulled the citations from iCite in November of 2022.

**Results:**

There were 2,391 articles published in 1,019 journals. The number of papers published by year increased from 56 in 2014 to 448 in 2021. The network had an average of 6.4 authors per paper, with this increasing by year from 5.3 in 2014 to 6.9 in 2021. The average journal impact factor for the overall network was 7.19, with a range from 0.08 to 202.73. The Oklahoma Shared Clinical and Translational Resources (OSCTR) network is a small world network with relatively weak ties.

**Conclusions:**

This study provides an overview of coauthorship in an IDeA-CTR collaboration. We show the growth and structure of coauthorship in OSCTR, highlighting the importance of understanding and fostering collaboration within research networks.

## Introduction

Coauthorship is a documentable form of research collaboration [1–7]. Social Network Analysis (SNA) is a method of analyzing coauthorship networks or relationships through graph theory [8,9]. Using SNA for coauthorship network is a well-established but relatively underutilized field [10,11]. Coauthorship is when two or more individuals work together on a single piece of written work. Co-authors typically collaborate on the content of the work and share responsibility for the final product. The practice of coauthorship is common in academia, where researchers often work together on projects and publish their findings in scholarly journals. Coauthorships, particularly in health research, have become standard practice, with the average number of coauthors increasing rapidly [4,12].

Currently, it is acceptable and, in fact, encouraged, and, often, necessary due to the complex nature of modern research, to have multiple coauthors [4,13]. In the late 1980s and early 1990s, coauthorship was suspect as a series of authors suggested that many authors had not put in an “appropriate” level of work into the product [1,14–16]. By the late 1990s it became clear that this “problem” was a trend, a pattern of increasing teamwork with the increased specialization and increased ability to collaborate with computer and internet advances [17]. Historically and in many social science fields coauthorship was considered unethical, it was believed to create unequal workload on a publication and it “watered down” the scientific literature [1]. However, coauthored papers are now expected in health publications particularly translational literature which often requires teams to understand appropriate methods for implementation [4]. In fact, as early as 1963 Price, in his seminar collection of lectures *Big Science, Little Science,* recognized that science was moving more toward teams [18]. He recommended looking at science scientifically or to study the output of science to understand and improve the implementation of science [18,19]. While he does not explicitly use social network analysis, he does recommend in another seminal work understanding networks of scientific papers [10]. He suggests that we try to picture the network obtained by linking published papers. Suggesting a visual representation that includes journals, countries, authors, or specific papers based on their positions within the map [10].

Institutional Development Award (IDeA) Networks for Clinical and Translational Research (IDeA-CTR) is now known as the IDeA Clinical and Translational Research Network (IDeA-CTRN) Award program. IDeA-CTRN was designed to expand the capability for clinical and translational research in IDeA-eligible states and to enhance competitiveness of researchers to obtain National Institutes of Health (NIH) funding. The CTRN goals include “(*1) expansion of existing infrastructure and human resources dedicated to conducting clinical and translational research, (2) enhancement of IDeA institutions’ and investigators’ ability to develop competitive clinical and translational research programs and (3) the coordination of collaborative clinical research activities across IDeA institutions and organizations*.”[20]

Coauthorship is a way to show effective collaboration. Several examples of coauthorship studies have been published from the 73 IDeA-CTRN (13) and Clinical and Translational Science Awards (CTSA) Program (60 awards that are similar in structure but not for IDeA states) [4,21–37]. Dozier *et al*. (2014) used a survey to determine emerging research collaboration and networks. They then used SNA to analyze intra and inter-departmental collaboration [23]. While they found this method useful, there were several important limitations – including a low response rate thus missing many research networks. Vacca and McCarty (2015) completed a study using SNA methods to understand an intervention used to enhance CTSA-based network members in cross-disciplinary team science in Florida [30]. The program matched researchers who had not published together based on one year of publications. They reported that some of these teams worked, although many did not, and that the closer the individuals were to each other in a network, the more likely they were to have a successful collaboration. Nagaranjan *et al*. (2011, 2015) showed that research networks in Arkansas grew and progressed using SNA methods [21,35]. However, that study chose to use research or funding networks, equally important collaborations, because they felt it represented emerging collaborations.

Publications point toward research trends and the contributions of an individual, organization, or network. Bibliometrics is a reasonable method to evaluate research trends over time qualitatively and quantitatively [38]. Bibliometric analysis has the advantages of being objective, is inexpensive, and takes very little time. Understanding how one CTR grows and succeeds is crucial to the nationwide NIH CTR initiatives. This study incrementally contributes to previous reports as it focuses on one IDeA-CTRN (Oklahoma), while others have focused on the whole nation CTSA program or used different data for their analysis, such as research or funding collaborations and surveys [22]. No SNA analysis has focused on a single IDeA-CTRN network, thus showing a gap in the literature. For this analysis, publications were readily available for all authors, but funding networks were not. Using SNA and bibliometric techniques, this study aimed to analyze the growth of one IDeA-CTRN program from inception to date.

## Methods

### Population

The data for this study was all publications from a cohort of clinical and translational scientists in Oklahoma. An individual becomes a member of the network by having been in a leadership role (core leaders, co-leaders, and staff), applied for or been funded as a pilot project recipient, having been recruited (funding being a part of the recruitment package), being a mentor to a pilot recipient, a member of the formal education programs (the Masters of Science in Clinical and Translational Research or the one-year Translation Practice Into Research program) or are a member of the Scholars program (a program for promising but unfunded early-stage investigators nominated by leadership). The Oklahoma Clinical and Translational Science Institute at the University of Oklahoma Health Sciences Center (OUHSC) is the academic home of the Oklahoma Shared Clinical and Translational Resources (OSCTR), which is awarded by the NIH to the OUHSC. Besides OUHSC, the Oklahoma Medical Research Foundation (OMRF), the University of Oklahoma Norman campus (OU Norman), the University of Oklahoma Tulsa campus (OUHSC Tulsa), and the Veterans Administration (VA) are partner organizations tied to OUHSC. Both VA and OMRF are on the same campus as OUHSC. In addition, the Oklahoma State University (OSU) and the Oklahoma State University Health Science Center serve as the other major academic health training centers in Oklahoma, training Doctor of Osteopathic Medicine, clinical psychologists, and public health professionals. Other partnering organizations include the Southern Plains Tribal Health Board (SPTHB), Cherokee Nation, Chickasaw Nation, and Laureate Institute for Brain Research. OSCTR serves as a catalyst to facilitate clinical and translational research; improve health for underserved and underrepresented populations living in rural areas; provide clinical and translational research training and infrastructure to junior investigators; expand opportunities for Oklahoma communities to participate in clinical and translational research; and improve the overall health of citizens, importantly Oklahoma’s rural, tribal, and minority populations.

### Data Collection

Data were collected through a search of PubMed from January 1, 2014 through December 31, 2021 in October 2022. We started by pulling an individual member’s name for the year they joined the network. Since the OSCTR program started in 2013, the first full year was 2014, and the final full year was 2021. In pulling that PubMed data, all articles published after joining OCSTR were included. Next, we searched by author name and affiliation in Oklahoma. We then hand-reviewed the papers to ensure that the author and affiliation were partners of OSCTR. Finally, we deleted those with more than 21 authors (n = 10).

For this study’s bibliometric portion, we pulled the citations in November of 2022. We used the National Institute of Health iCite, a tool to access several bibliometrics measures for papers. Searching the PubMed IDs for articles of interest in iCite allowed us to understand the citations for each article. We used the number of citations per year. The impact factor was determined through Journal Citation Reports annual publication by Clarivate Analytics.

### Method of Analysis

This study uses SNA to study a coauthorship network. SNA uses patterns of relationships among people in groups. They are helpful for examining the social structure and interdependencies (or coauthorship patterns) of an organization or group of nodes that are the basic unit of a network. Nodes, in this case, represent authors. In the visualizations for this study, node size is proportional to the number of publications. Edges or ties connect two nodes in the network and indicate a coauthorship with the number of coauthored papers between the two nodes or authors represented with bigger lines representing more coauthorships. Visualization of the coauthorship network is essential to complement the analysis [4]. Network measures in SNA examine the overall properties and structures of the entire network providing insights into the network’s organization. In contrast, author measures focus on individual nodes or authors within the network, assessing their attributes like degree centrality or betweenness centrality, which help identify influential or pivotal nodes in the network. All network analysis was completed using UCINET [39].

### Network Measures

This overall network is undirected, meaning all edges are bidirectional. Nodes represent the number of unique authors in the network. Ties are the number of connections or publications between authors. We will discuss several measures of SNA, including *density*, *average degree*, *average distance*, and *clustering coefficient C*. The *density* of a network is the total number of edges divided by the total number of possible edges. It is a widely used measure that reflects the level of cohesion among network authors or the extent to which authors collaborated with other authors in the network. The *average degree* counts the number of connections for any given node or author. The higher the *average degree*, the more connected the networks or the average number of author collaborations. *Average distance* is the average number of steps along the shortest paths for all possible pairs of network nodes. It is a measure of the efficiency of a network. *Clustering coefficient C* measures how many of the authors connected to a given author are also connected to each other, which is expressed as a proportion of the total possible connections. The overall *clustering coefficient C* is the average across the network. Where *density* tells an author’s connection to the network, the *clustering coefficient C* tells how well connected the various authors in the network are. A high *clustering coefficient C* and low *density* can indicate lots of loosely connected small groups.

Small world networks or the small worldness of a network is a category of mathematical graph measuring coauthors who are not neighbors of one another. The neighbors of any given author are likely to be neighbors of each other and most authors can be reached from every other author by a small number of steps. Small world networks underscore the interconnectedness of the network, facilitating efficient communication, information flow, and the potential for unexpected opportunities and collaborations. A measure of three or greater suggests the network is a small world network.

Network measures in social network analysis focus on properties of the entire network structure, while actor (or author) measures focus on individual nodes' characteristics and their roles within the network.

### Author Measures

Besides network measures, we measured characteristics of each author in the network. Author measures in SNA allow researchers to identify key individuals within a network, helping to pinpoint influential actors, potential leaders, or information brokers but also where we find these individuals within the network. We used the whole 2014-2021 network. *Degree* represents authors who are the most connected or popular in the network. *Degree* is a basic measure of centrality in SNA. *Degree* is defined by the number of direct ties a particular author has in a network [40]. *Degree* is a measure of highly connected authors and eventually reflects those authors having more direct contact and thus adjacency with other authors in a network. *Closeness* represents an expanded degree of centrality by focusing on how close an author is to all other nodes in the network. For an individual author, it represents to what extent an author is in proximity in the network to other authors. People with high *closeness* are more important in the distribution of information or are the shortest path between one author and another. *Betweenness* represents people who are informal power brokers or bridges between clusters or groups of other authors. *Betweenness* is obtained by determining how often a particular author is found on the shortest path between any pair of nodes in the network. *Betweenness* views an author as favored in that the author falls on the shortest path between other pairs of authors in the network.

### Bibliometric Measures

Finally, we completed a limited bibliometric analysis of the OSCTR network publications. Bibliometric analysis is a quantitative method used to evaluate and study patterns, trends, and relationships within academic literature, including publications, citations, and authorship, to gain insights into the scholarly landscape. For this study we looked at overall publication trends, but also journal impact factor and article citation. These measures are used to show the quality of network’s publications [41,42].

## Results

### Bibliometric Analysis

The number of individuals in the network grew from 48 in 2014 to 331 in 2021 (Table 1). Among the 2,391 articles produced by the network partners, the total number of papers published by the year increased from 56 in 2014 to 449 in 2021 (Table 1). In this network, single, authored publications were rare, with the highest number being three in 2016 (Table 1) and only ten in the whole eight years. The mean number of authors increased from 5.3 in 2014 to 6.9 in 2021, despite eliminating publications with more than 21 authors (*n* = 10). Among the 376 OSCTR network members, 268 (71%) published at least one article over the whole period of study. There was an increase in authors from 24 in 2014 to 178 in 2021. There was a dramatic increase in the number of ties (collaborations) from 14 in 2014 to 374 in 2021. There were few ties (coauthorships) in 2014, suggesting that early on, many articles were published by a small number of people without a significant number of coauthorships within the network. The number of publications and authors increased yearly with a slight drop during the early stages of the COVID-19 pandemic in 2020 (Table 1).

**Table 1.** Oklahoma Shared Clinical and Translational Resources network analysis measures summary by year 2014–2021

	2014	2015	2016	2017	2018	2019	2020	2021	Total
Number in network	48	124	197	229	258	295	321	331	376
Total papers	56	172	277	292	374	368	403	449	2,391
Single author papers	0	1	3	1	2	1	1	1	10
Collaborative papers	56	171	274	291	372	367	402	448	2,381
Mean authors per paper	5.3	5.7	5.9	5.9	6.5	6.8	6.8	6.9	6.4
Nodes (authors)	24	66	111	138	151	151	160	178	268
Ties (coauthorships)	14	62	174	252	250	344	358	374	1,214
Average degree	0.583	0.939	1.568	1.826	1.656	2.278	2.237	2.101	4.530
Density	0.033	0.014	0.014	0.013	0.011	0.015	0.014	0.012	0.017
Average distance	1.0	1.679	3.071	4.235	4.258	4.456	5.090	5.173	3.336
Clustering coefficient C	1.333	0.702	0.480	0.365	0.413	0.437	0.430	0.363	0.250
Main component									
Nodes (authors)	*	*	34	64	46	80	83	105	212
Ties (coauthorships)	*	*	114	204	138	290	312	344	1,204
Small worldness	*	*	4.155	5.671	4.309	7.192	6.232	8.515	9.041

* No main component present; #articles with 21 or more authors were delete.

Among the 2,391 articles published in 1,019 journals, those with the most were in Geroscience (64), PLoS One (37), Scientific Reports (30), International Journal of Environmental Research and Public Health (23), Gynecological Oncology (22), American Journal of Hematology (21), and American Journal of Preventive Medicine (20) (Table 2).

**Table 2.** Oklahoma Shared Clinical and Translational Resources top 15 publications by journal by year 2014–2021

Journal	2014	2015	2016	2017	2018	2019	2020	2021	Total	Impact Factor 2022^1^
Geroscience				7	7	16	7	12	49	7. 581
PLoS One	2	4	4	5	11	2	5	4	37	3. 752
Sci Rep		1	3	3	3	4	9	7	30	4. 997
Int J Environ Res Public Health		1	2		1	2	7	10	23	4. 614
Gynecol Oncol		1	5	5	5	3	2	1	22	5. 304
Am J Hematol		3	4	3	1	6	3	1	21	13. 268
Am J Prev Med		11	1	2		2		4	20	6. 604
Nicotine Tob Res	1	1	3	4	2	2	3	3	19	5. 825
Front Immunol				1	5	2	6	5	19	8. 787
Oncotarget		5	7	4		1	1		18	0. 966
Addict Behav				2	6	6	2	2	18	4. 591
Int J Mol Sci					2	3	6	6	17	6. 208
Nutrients				2	2	3	3	6	16	6. 706
Cancers (Basel)					2	3	4	7	16	6. 575
Drug Alcohol Depend			3	1	1	4	3	4	16	4. 852

1 Journal Impact Factor (JIF).

The average impact factor for the overall network was 7.19, with a similar average impact factor in most years (Table 3). The journal impact factor that stands out includes Lancet (2) at 202.731, New England Journal of Medicine (10) at 176.082, and Journal of the American Medical Association (3) at 157.357 (data not shown). The most cited article from the network was entitled “Mental Health and the Covid-19 Pandemic” by Pfefferbaum and North (2020) in the New England Journal of Medicine with 844 citations (data not shown) [43].

**Table 3.** Oklahoma Shared Clinical and Translational Resource publications citation count, mean citation per articles, and maximum number of citations 2014–2021

	2014	2015	2016	2017	2018	2019	2020	2021	Total
**Citations**									
Citation count	121	580	913	1,123	1,458	1,779	2,635	1,643	10,250
Mean citations per article per year	2. 15	3. 37	3. 29	3. 84	3. 90	4. 83	6. 54	3. 66	4. 29
Maximum number of citations for one article	18	26	62	37	51	121	844	91	844

### Network Analysis

**Figure 1.**
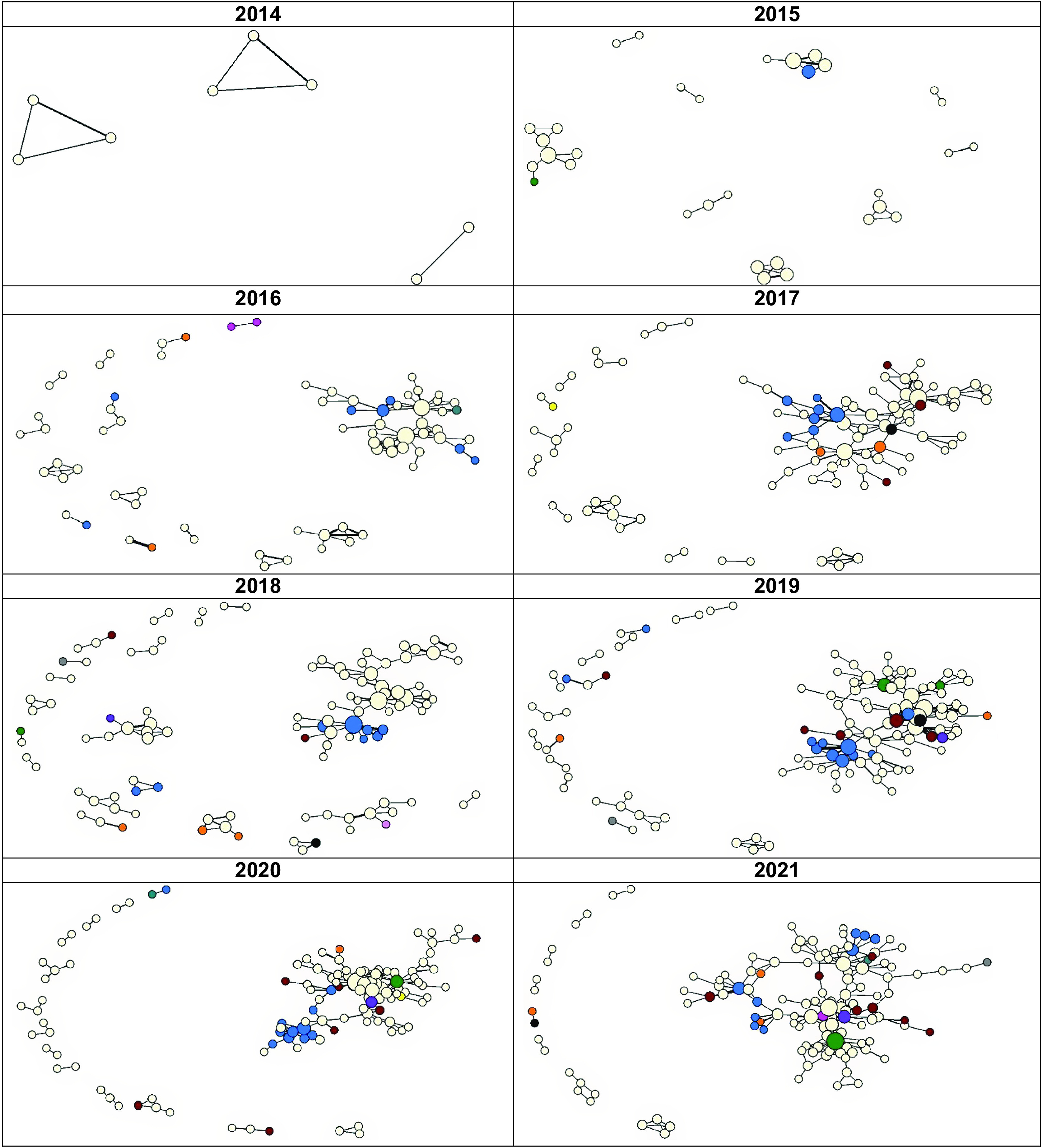
Evolution of the Oklahoma Shared Clinical and Translational Resources network using social network analysis 2014–2021.

The average network *degree* and *average distance* both increased (Table 1) while the *clustering coefficient C* decreased suggesting a decrease in how many of the authors connected to a given author are also connected to each other. The coauthorship network did not have a main core (large and connected group of coauthors) until 2016, which then evolved into a strong core from 34 authors in 2016 to 105 in 2021 (Table 1; Fig. 1) with a total of 212 in the main component of the network. Additionally, the number of ties (coauthorships) in the main component increased from 34 in 2016 to 344 in 2021, totaling 1,204 in the main core (data not shown).

Among the 376 authors, 288 (76.6%) were from OUHSC, 18 (4.8%) were from OMRF, 18 (4.8%) were from OU Norman, and 13 (3.5%) were from OSU. Among the 288 OUHSC authors, 193 (67.0%) were from the College of Medicine, 28 (9.7%) from the College of Public Health, 24 (8.3%) from the College of Allied Health, 17 (5.9%) from the College of Dentistry, and 15 (5.2%) from the College of Nursing (data not shown). The remaining 11 were from the College of Pharmacy (8) and the Dean McGee Eye Institute (3) (data not shown).

### Growth of the Network

A visualization of the growth of the network is shown in Fig. 1. The visualization of the network by year clearly shows growth in the network; the number of authors and the coauthorship ties is shown to increase yearly. Additionally, as shown in the visualization below the growth of the main core of the network has expanded each year beginning in 2016. The small worldness of the main component of the network increased from 4.155 in 2016 to 8.515 in 2021, suggesting this network was a small-world network.

**Figure 2.**
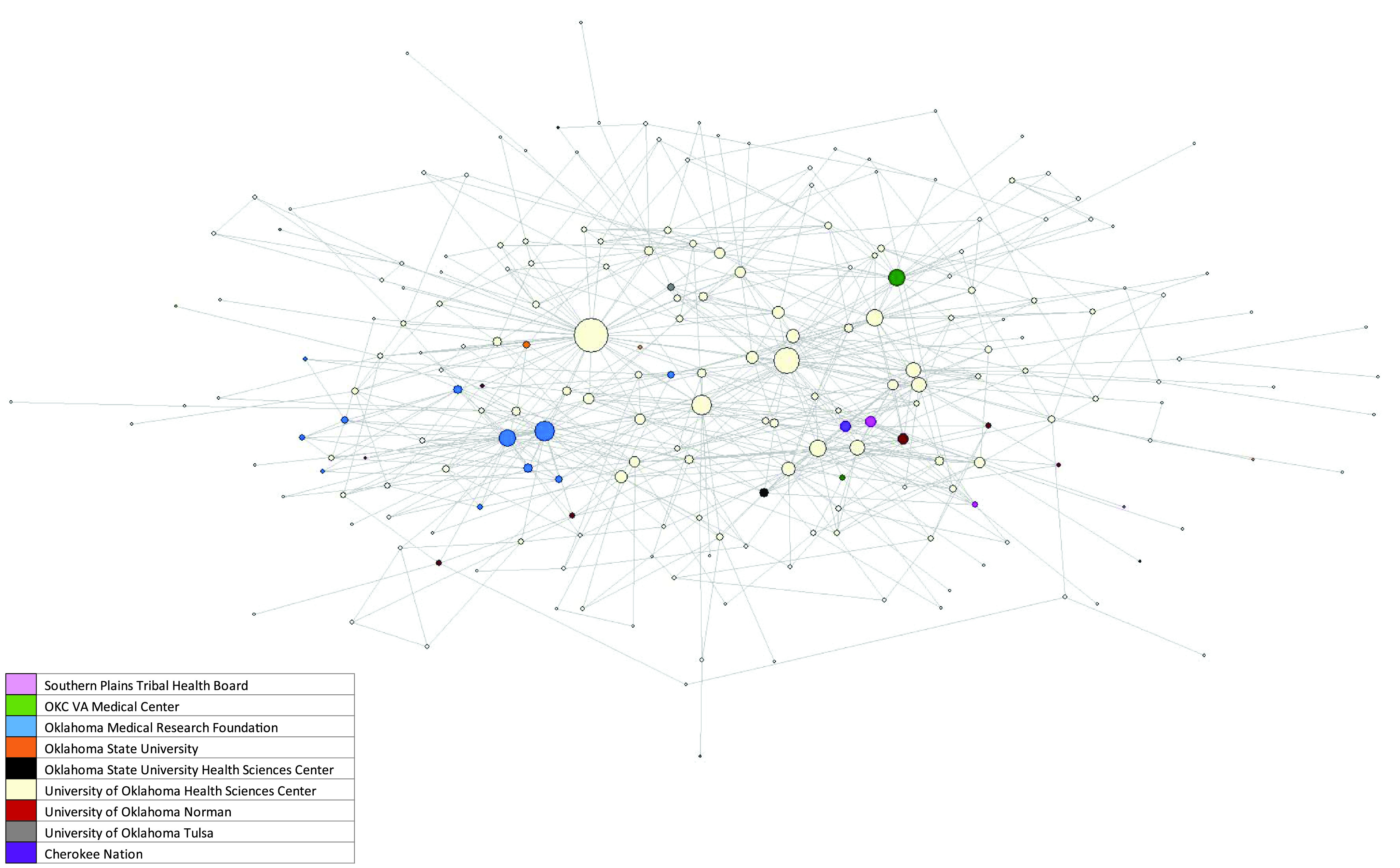
Oklahoma Shared Clinical and Translational Resources network main core using social network analysis 2014–2021.

**Figure 3.**
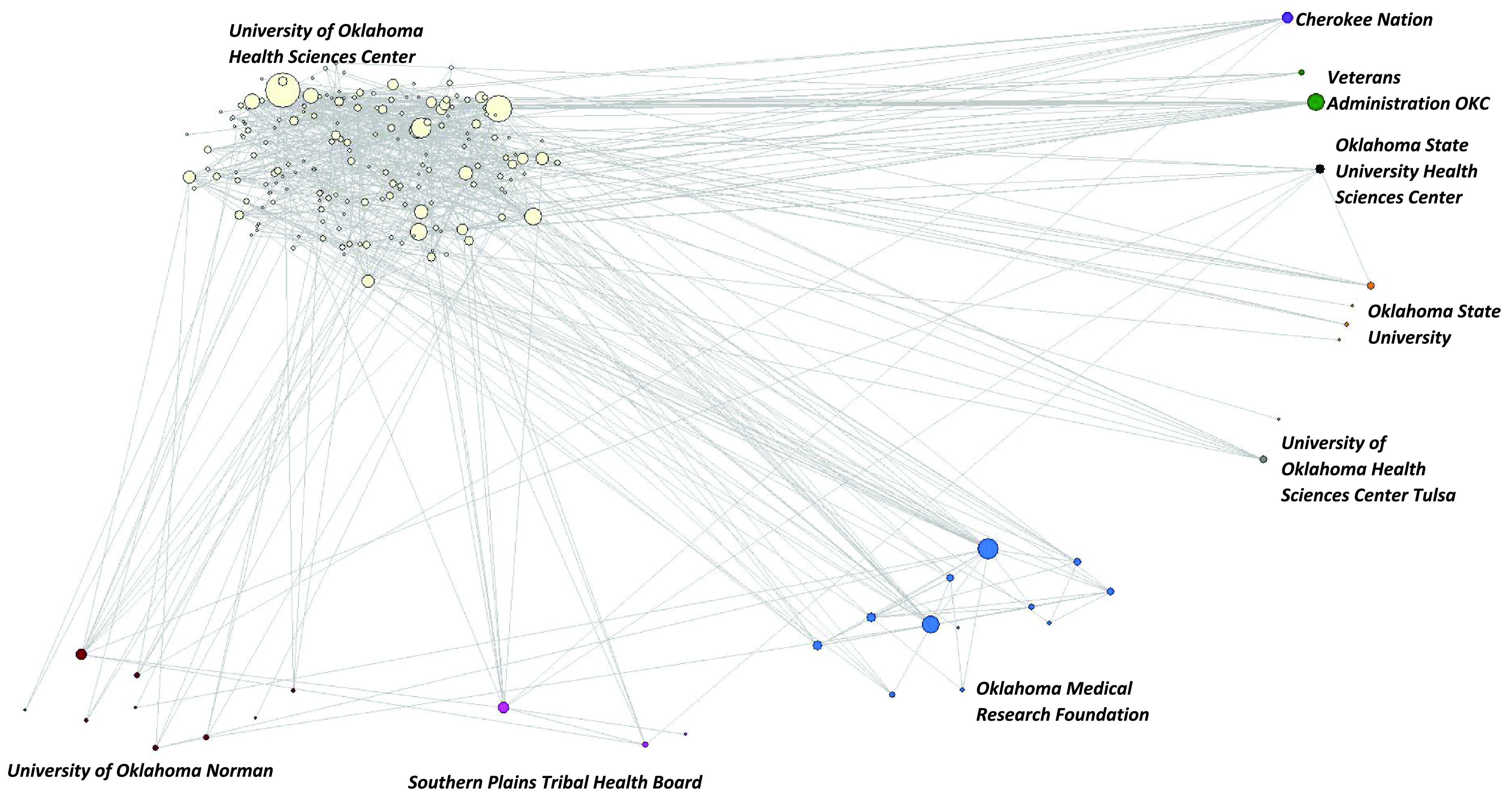
Oklahoma Shared Clinical and Translational Resources network main vore grouped using social network analysis by organization 2014–2021.

**Figure 4.**
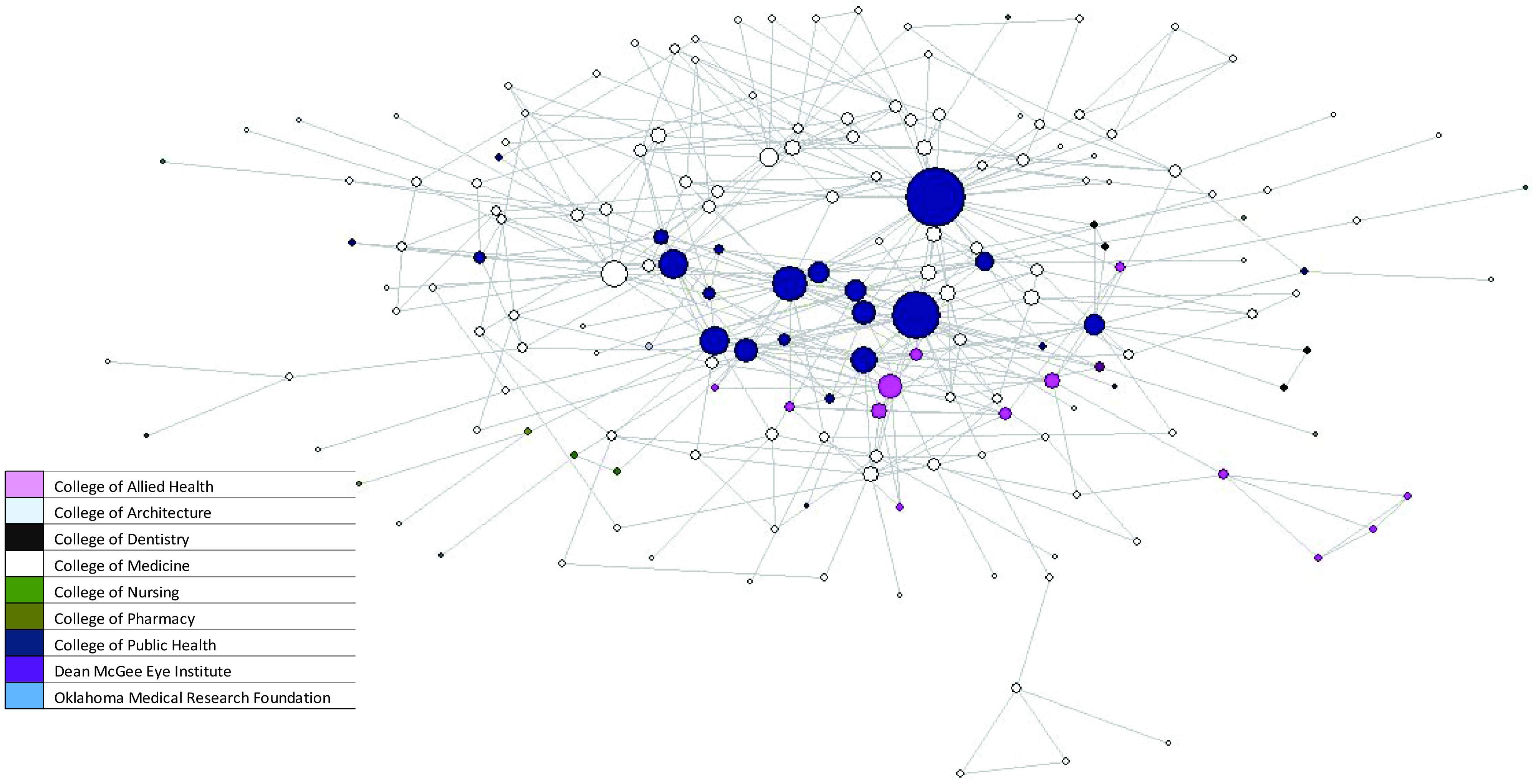
Oklahoma Shared Clinical and Translational Resources 2014–2021 University of Oklahoma Health Sciences Center network main core using social network analysis color by college.

Visualization of the network shows that it grew dramatically in the first three years and that there was a strong main core from that date on. Interestingly, no matter the year after 2017, the main core shows a clustering of OMRF and OUHSC in the main component (Fig. 1 and Fig. 2). Fig. 3 shows the main core disarticulated by organization. The largest group was OUHSC (182), followed by OMRF (11), OU Norman campus (9), Oklahoma State University (4), SPTHB (3), OUHSC Tulsa (3), and Cherokee Nation (1). Eight members of the OUHSC group were only connected to the main component through a non-OUHSC member. Among the 174 nodes that from OUHSC and connected (Fig. 4) to the main component is the College of Medicine (120), College of Public Health (27), and College of Allied Health (13). The College of Medicine, as well as the smaller represented colleges (Dentistry (4), Pharmacy (4), Nursing (5), and Architecture (1)) tended to be on the periphery of the network.

Another significant result noted from Fig. 3 and Fig. 4 is the links with partnering organizations. There were at least two sub-cores on OUHSC (cream) and one OMRF (blue). In addition, OMRF had a very tight and strong sub-core with considerable strength of ties not only with OUHSC but also among themselves (Figs. 3 and 4). Additionally, visualization showed the importance of specific OUHSC members to partner organizations, such as the VA, Cherokee Nation, OU Norman, OUHSC Tulsa, and the SPTHB (Figs. 3 and 4).

### Author Analysis

*Degree* shows authors who are the most connected in the network. It is used for finding individuals who are likely to hold the most information or individuals who can quickly connect with the wider network. Among the top twenty authors in *degree*, 17 (85%) were from OUHSC, two (10%) from OMRF, and one (5%) no longer works for the University (data not shown). Among the 17 from OUHSC, 12 (70%) were from the College of Public Health, three (18%) were from the College of Medicine, one (5%) was from the College of Allied Health, and one is no longer with the University (data not shown).

*Closeness* represents an expanded degree of centrality by focusing on how close an author is to all other nodes in the network. It is used to find the best individuals to influence the entire network quickly. Among the top 20 (22 due to ties) authors, 20 (91%) were from OUHSC, and two (9%) were from OMRF (data not shown). Among the 20 from OUHSC (13), 11 (55%) were from the College of Public Health, seven (35%) were from the College of Medicine, and two (10%) were from the College of Allied Health (data not shown).

*Betweenness* represents people who are informal power brokers or bridges between clusters or groups of authors. They often bridge different groups; for example, they bridge some of the smaller organizations, such as the VA and SPTHB. This means that these individuals manage the information flow around the network. Among the top twenty in *betweenness*, 17 (85%) were from OUHSC and two (15%) were from OMRF (data not shown). Among those from OUHSC, 12 (71%) were from the College of Public Health, four (24%) were from the College of Medicine, and one (6%) has left the university (data not shown; does not add to 100% due to rounding).

To determine some of the most influential authors in the network, we looked at the overlap between d*egree* (most connected) and *betweenness* (bridgers). Fourteen authors were in the top 20 of both *degree* and *betweenness*. Among those 14 authors, 12 were from OUHSC and two were from OMRF. Among the 12 from OUHSC, eight were from the College of Public Health, two were from the College of Medicine, one was from the College of Allied Health, and one had left the university (data not shown).

## Discussion

The OSCTR coauthorship network showed some significant growth as well as an interesting network structure. It is clear from this study that there was an increase in not only the number of authors but also their coauthorships suggesting more collaborations. The OSCTR network is a small world network that has relatively weak ties with only 1.7% of the network that can be connected via a coauthorship. Weak ties, it has been suggested, lead to greater collaboration or more collaboration will occur in networks with weaker ties [44,45]. In practical terms, a high number in small worldness suggests that information or interactions can spread efficiently and rapidly within the network, which can be advantageous for communication, diffusion of innovations, or collaboration.

The OSCTR network evolved into a large network that still has opportunities, for example, 111 network members having no authorship in the main component. Moreover, the number of *average degree*-ties (or coauthorships) divided by nodes (or authors) showed an increase. In other words, there were more ties for each author. The two primary organizations (OUHSC and OMRF) dominate the network, particularly OUHSC with their large number of authors, articles, and being the larger organization (around 5,000 compared to around 450). It is important to note that OMRF is a private nonprofit research institution on the OUHSC campus. The OMRF sub-core was a tightly connected sub-core that was also connected through multiple individuals to the OUHSC sub-core. The OUHSC main component was large and impressive with a variety of colleges represented. However, we clearly have opportunities among our partner programs to increase coauthorship even further. Additionally, the OUHSC authors have bridges to these other organizations including OU Norman, OSU – both the main campus and the health science center campus, SPTHB, OUHSC Tulsa, Cherokee Nation, and Texas Biomedical Research Institute. The OSCTR partner organizations have shown to be effective at the goal of OSCTR and all Clinical and Translational programs to “*promote partnerships and collaborations to facilitate and accelerate translational research projects locally, regionally and nationally* [46]*.”*

In the late twentieth century coauthorship networks began to be analyzed through social network analysis [1–8,10]. Early researchers noted that coauthorship networks often contained a small number of influential authors and many peripheral authors [7]. This is very similar to what we saw in this network where there are some strong authors in the center with many publications as well as collaborations. Both the visualization of the network as well as the *degree*, *betweenness,* and *closeness* of the network show that public health is a critical part of the OSCTR network. In our author analysis, we see that among the top ten authors by *degree* 50% were from the College of Public Health. In *betweenness* and *closeness,* seven of the top ten (70%) were from the College of Public Health. Additionally, it must be noted that OSCTR offers no-cost statistical, methodological, analytical, grant, and manuscript assistance to those in OSCTR. This finding has also been shown by Hunt *et al*. (2012) for the Indiana Clinical and Translational Sciences Institute (ICTSI) [36]. The ICTSI, with only three years of data, showed that collaborations increased, every program was represented, and biostatistics, bioethics, and the project development team were at the center of the network [36]. This is similar to what we found as publications often need a statistician and may require a methodologist to analyze the strengths and weaknesses of the research methods. Public health professionals who receive broad training in biostatistics and epidemiological methods are an important component in these clinical and translational research teams.

The OSCTR network, visually, became more connected through time, though this was not shown through the *density*, which is decreasing through time; however, there was an increase in *average degree* over time. Other research has shown the growth of networks like what we see in the OSCTR network [47]. This latter study used a coauthorship network of continuous quality improvement publication from the Australian Indigenous primary healthcare. The growth of that network was very similar moving from a small collaboration of three authors to one with dozens of authors with several sub cores, albeit over a longer time -18 years.

This study has several strengths including clear collaborations as shown through coauthorship using standard SNA measures and a relatively long number of years (8) of the network. Coauthorship networks being analyzed through SNA is a well-established and easily replicable method. Additionally, this study used bibliometric attributes of the coauthorship network to look at the quality of the publications as well as the quantity and connections shown by using SNA.

Despite these strengths, there are several limitations of this study. First, we only used PubMed to find articles – thus possibly missing some articles. Second, disambiguation of names was necessary and completed via hand review by the author (JEC) it was not completed with duplicate review. Third, because network membership does not fully depict all collaborations as those that are not OSCTR partners would not be represented. While this is a significant issue, the goal of this project was to look at the OSCTR network of publications. Fourth, we do not know the quality or longevity of the collaboration.

Overall, this study showed the growth, development, and structure of one IDeA-CTRN program in Oklahoma. While that growth has been dramatic, we see a slight leveling off over the last three years. We expect this trend to continue. Moreover, this study shows the program management the width and depth of author collaboration as well as the potential for more and stronger collaboration. Li *et al*., showed in 2018 that early coauthorship with successful scientists predicted future success [48]. Other networks have also shown the importance of studying these networks [49–52]. This study shows who, in the OSCTR context, are successful based on joint publications and those who will encourage collaborations with less experienced scientists. This allows us to understand ways to foster collaboration. This research broadens our understanding of the secondary effects exerted by clinical and translational science institutes on the scientific networks of researchers. This approach, centered on one network, could serve as a valuable tool for assessing the influence of collaborative science initiatives such as OSCTR within a university setting.

## Data Availability

These data are downloaded from PubMED.
